# Amputation of the Thigh, for Caries of the Knee Joint

**Published:** 1838-04-25

**Authors:** Thomas Harris


*Amputation of the Thigh, for Caries of the Knee Joint, by* THOMAS HARRIS, M. D.

Peter C——, aged 22, a shoemaker by trade, was admitted into the house in the winter of 1836, for an injury to his knee from a fall. He had had pain in it for some months previous, but not so as to prevent his attending to his work. After staying in the house about six weeks, he was enabled to resume his work. On the 20th of December, 1837, he was re-admitted, the knee much swollen, and the bones carious, a probe passing readily into the joint. He was put under an alterative treatment, his general health being bad. The limb was placed in a carved splint, and kept at perfect rest. The disease, however, progressed; his constitution was suffering severely, and, upon a consultation, amputation was determined upon.

*February* 28th, 1838. Dr. HARRIS performed the common circular operation on the thigh—present, Drs. Randolph and Norris. The usual dressings were applied, and the wound healed almost entirely in three weeks. During the cure, the patient was affected with violent neuralgia of the stump, which was relieved by the free use of anodynes. Dissection of the knee showed the cartilages almost removed by ulceration, the head of the tibia diseased, and the condyles of the femur bare.

